# “Suicide Clusters: Analysis of a Sample of Completed Suicides in Spain”

**DOI:** 10.1192/j.eurpsy.2024.1620

**Published:** 2024-08-27

**Authors:** A. M. G. Alvarez, J. J. M. Jambrina, I. F. Arias, L. P. Gómez, N. A. Alvargonzalez, C. P. Miranda

**Affiliations:** Psychiatry, Hospital San Agustin, Aviles, Spain

## Abstract

**Introduction:**

“Cluster suicides,” also known as “suicide clusters,” refer to a phenomenon in which a series of suicides occur within a specific community, group, or geographic area within a relatively short period of time. These suicides often appear to be interconnected, either through imitation or contagion, and may involve individuals who have some form of social or emotional connection to each other.

**Objectives:**

- Understanding the definition and characteristics of cluster suicides.
- Analyzing common risk factors and triggers in cluster suicide cases.
- Evaluating prevention and support strategies for affected individuals and communities.

**Methods:**

We conduct an analysis of this concept based on a sample of suicides that occurred in a Spanish region over an 8-year period (2015-2022).

We will Analyzethe following aspects:

- Definition and characteristics of cluster suicides.
- Risk factors contributing to the occurrence of cluster suicides.
- Examples of real cases or case studies illustrating this phenomenon.
- The role of imitation and contagion in cluster suicides.
- Prevention and support strategies, including education on warning signs and access to mental health services.
- The impact of media coverage and how it can amplify the contagion effect.
- Measures to reduce access to lethal means of suicide.

**Results:**

We will discuss about the results found:

- Definition and characteristics of cluster suicides.
- Risk factors contributing to the occurrence of cluster suicides.
- Examples of real cases or case studies illustrating this phenomenon.
- The role of imitation and contagion in cluster suicides.
- Prevention and support strategies, including education on warning signs and access to mental health services.
- The impact of media coverage and how it can amplify the contagion effect.
- Measures to reduce access to lethal means of suicide.

**Conclusions:**

**The main conclusions of our presentation are :**

- The importance of recognizing cluster suicides as a real and concerning phenomenon.
- The need to address specific risk factors and triggers in affected communities.
- The effectiveness of prevention and support strategies in reducing cluster suicide cases.
- The importance of promoting media responsibility in suicide coverage.

BIBLIOGRAPHY

1. **Cluster Suicides: A Critical Review and Theoretical Framework”** (2019) - Este estudio proporciona una revisión crítica de la literatura sobre cluster suicides y presenta un marco teórico para comprender mejor este fenómeno
2. **“Clusters of Suicides and Suicide Attempts: Identification, Prediction, and Prevention”** (2016) - Aunque este estudio no se centra exclusivamente en España, ofrece información sobre la identificación y prevención de clusters de suicidio que puede ser relevante.
3. **“Epidemiology of Suicide in Spain, 1981–2008”** (2012) - Proporciona una visión general de la epidemiología del suicidio en España, lo que podría ayudar a contextualizar los estudios específicos sobre clusters.

**Disclosure of Interest:**

None Declared

